# Supplementary figures and images for: Forward genetics in Wolbachia: Regulation of Wolbachia proliferation by the amplification and deletion of an addictive genomic island
Source: PLoS Genet. 2021 Jun 18;17(6):e1009612. doi: 10.1371/journal.pgen.1009612 (PMC8244876; doi:10.1371/journal.pgen.1009612)

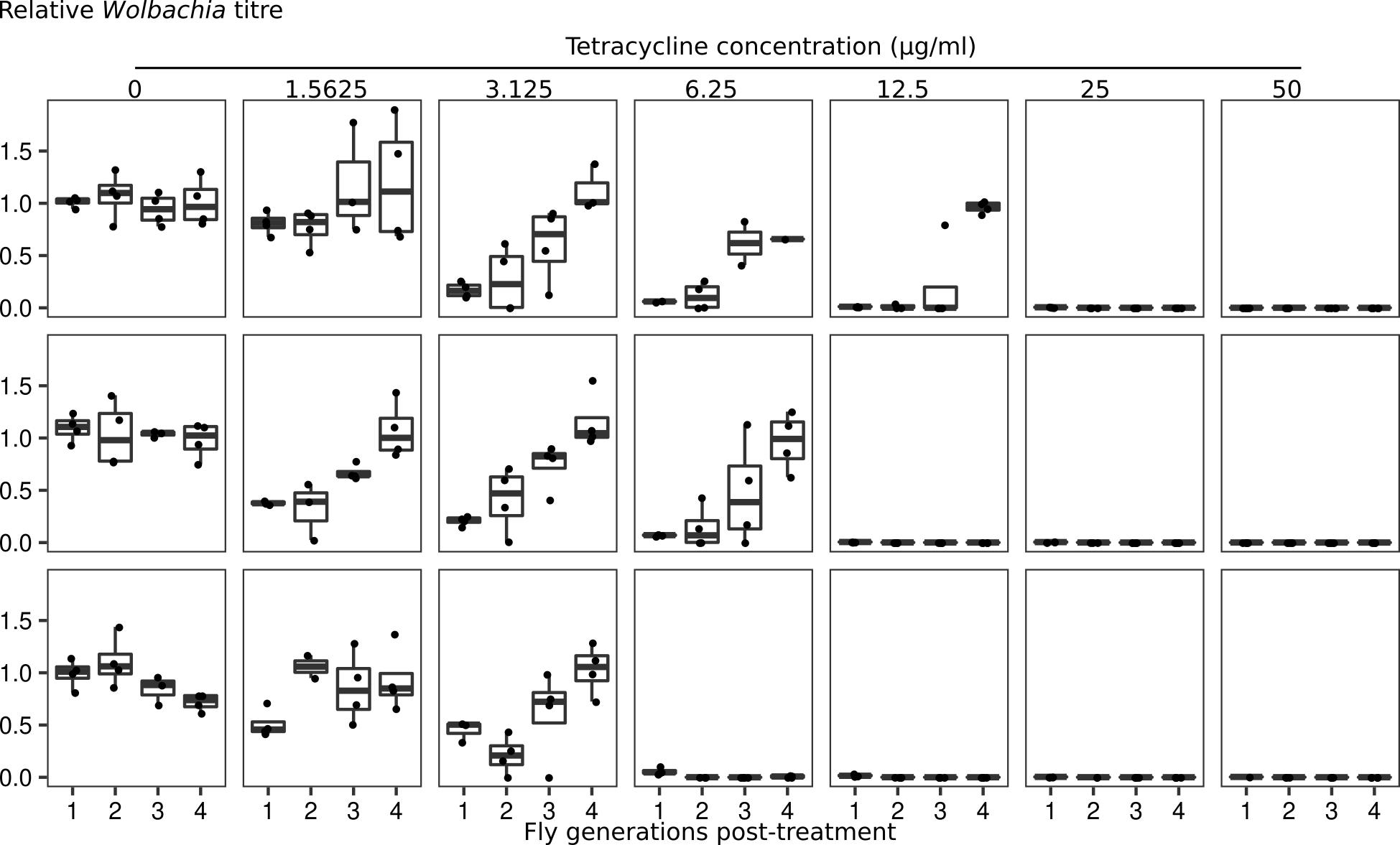

Supplement: S1 Fig — Relative Wolbachia titres of the progeny of tetracycline-treated flies. wMelCS_b-carrying females laid eggs in food containing varying doses of tetracycline. The progeny of three females were used to set up the experiment. At the first generation, four females were randomly selected for egg-laying in antibiotic-free fly food and Wolbachia titre was measured using qPCR. Titres of untreated females were used to normalize the qPCR results. The progeny of a female with the median titre was used to set up the next generation. Wolbachia titre in the F1 was significantly determined by the concentration of the antibiotic (p < 0.001 for all doses compared with control at generation 1), but recovered to normal within four fly generations (p > 0.05 for all doses compared with control at generation 4). (TIFF) [file pgen.1009612.s001.tiff]

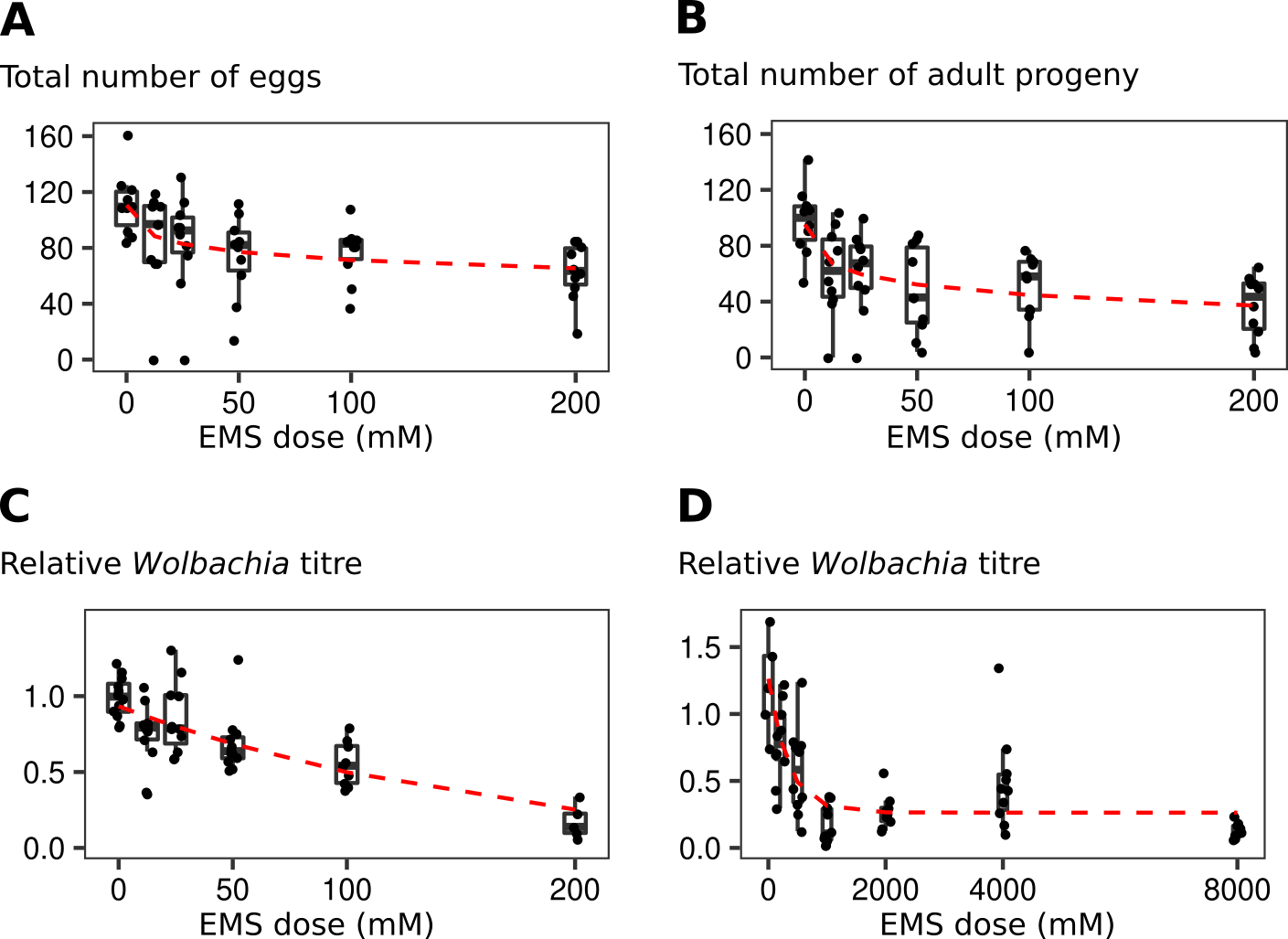

Supplement: S2 Fig — The total number of eggs (A) and adults (B) from females treated with varying doses of EMS. The reproductive output of 10 females was determined in the first ten days after EMS treatment by daily transferring females to new vials for egg laying. Females fed on a sucrose solution served as controls. Each dot represents the total number of eggs (A) or adults (B) laid by individual females during ten days. The effect of EMS on the reproductive output of females was estimated using a non-linear model and was highly significant (p < 0.001 for both numbers of eggs and adults per female). (C and D) Wolbachia titres in the F1 progeny of females treated with varying EMS doses. Wolbachia titre was quantified on individual females (n = 5–13 per dose), after laying eggs for three days. Wolbachia titres were normalized against the titres of untreated females. Dashed red lines represent the mean value predicted using non-linear models. The effect of EMS on Wolbachia titres in the next generation was highly significant (p < 0.001 for both panels). (TIFF) [file pgen.1009612.s002.tiff]

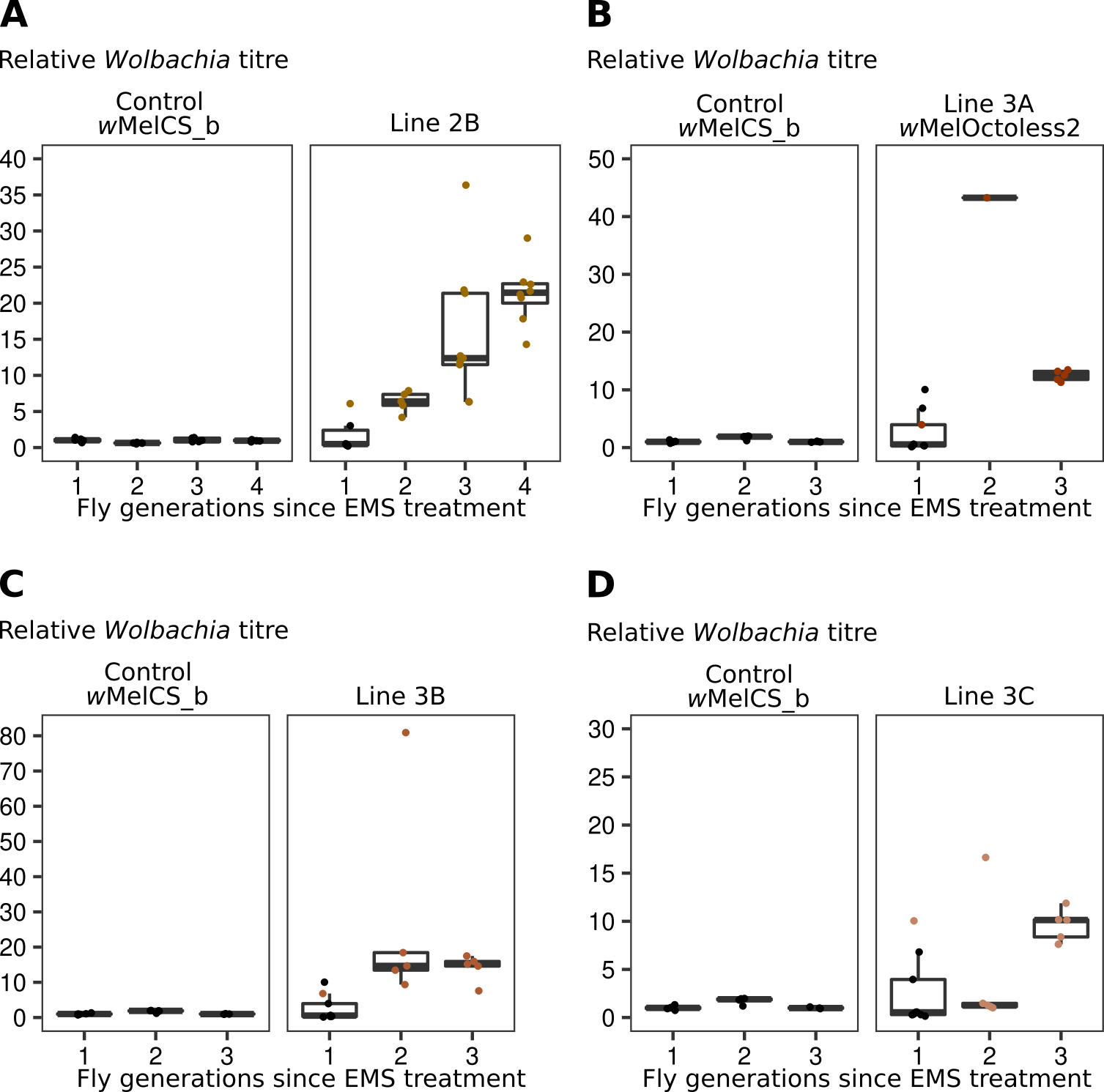

Supplement: S3 Fig — (A-D) Relative Wolbachia titres in a control (wMelCS_b) and EMS-treated D. melanogaster lines. Flies to set up the next generation was selected as described for Fig 1. Line 2B was isolated in the same batch as Line 2A (wMelOctoless) and they may be not independent. Likewise, Lines 3A (wMelOctoless2), 3B, and 3C were also isolated in a same batch. (TIFF) [file pgen.1009612.s003.tiff]

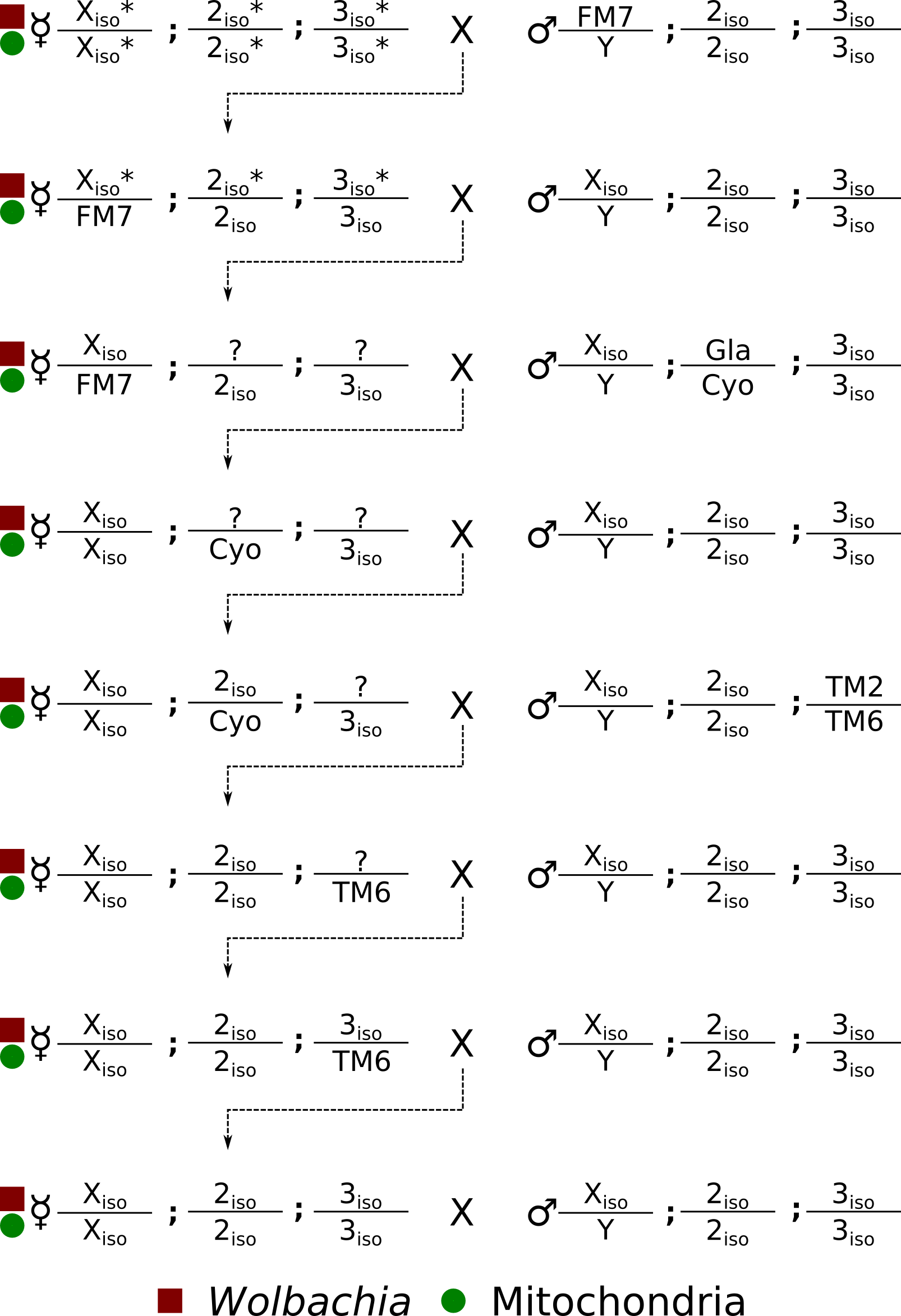

Supplement: S4 Fig — The first, second and third chromosomes of flies carrying wMelPop2, wMelOctoless, and wMelOctoless2 were replaced through the use of balancer chromosomes. Wolbachia infection (and also mitochondria) was kept in the stock by crossing females with Wolbachia with indicated males. The mitochondria are only shown in females because of its strictly maternal transmission. All males were free of Wolbachia infection. Dashed lines indicate the genotype selected from the previous cross. Virgin female in the first cross were considered mutant in all chromosomes (*), for illustrative purposes. Question marks (?) represent recombined chromosomes. (TIFF) [file pgen.1009612.s004.tiff]

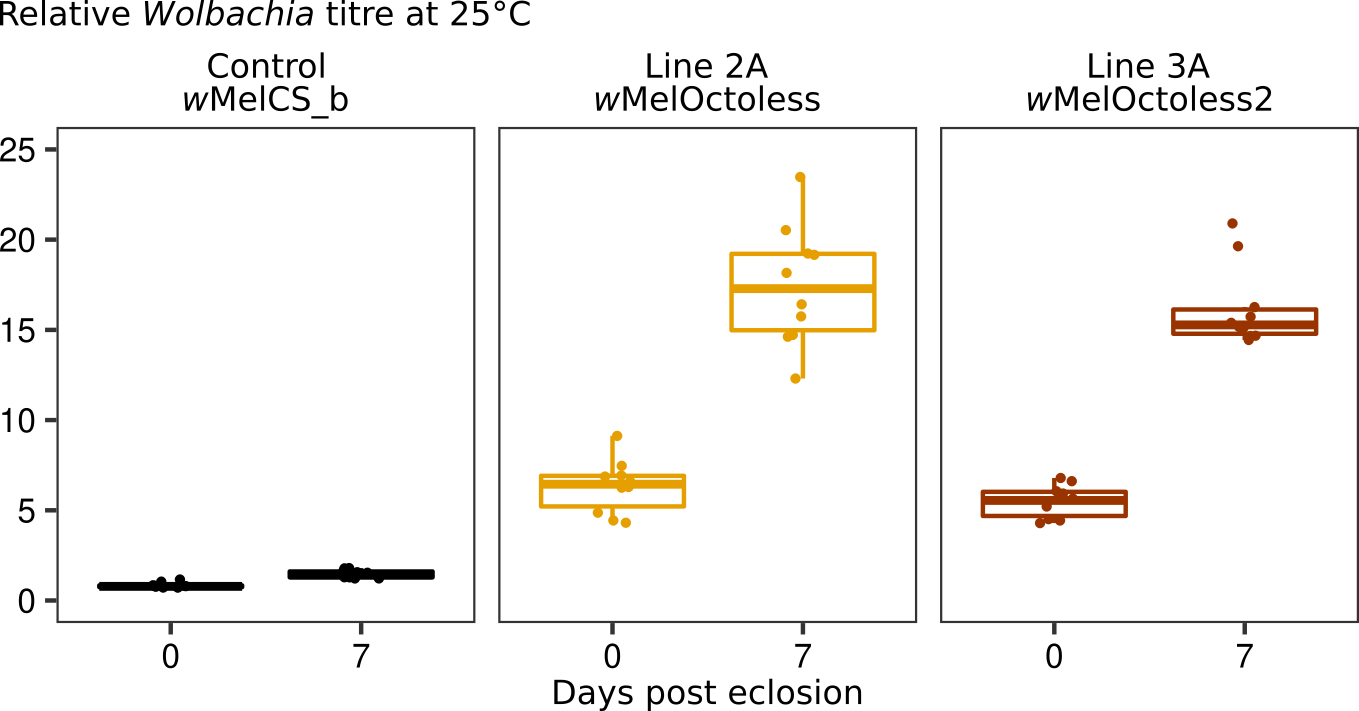

Supplement: S5 Fig — Relative Wolbachia titres in D. malanogaster males carrying wMelOctoless and wMelOctoless2 at 0 and 7 days post adult eclosion, at 25°C. This experiment was set-up as described in Fig 1. Relative Wolbachia titre was determined using qPCR and normalized to that of 0–1 days-old wMelCS_b-infected males. Each dot represents the relative titre of a single male. (TIFF) [file pgen.1009612.s005.tiff]

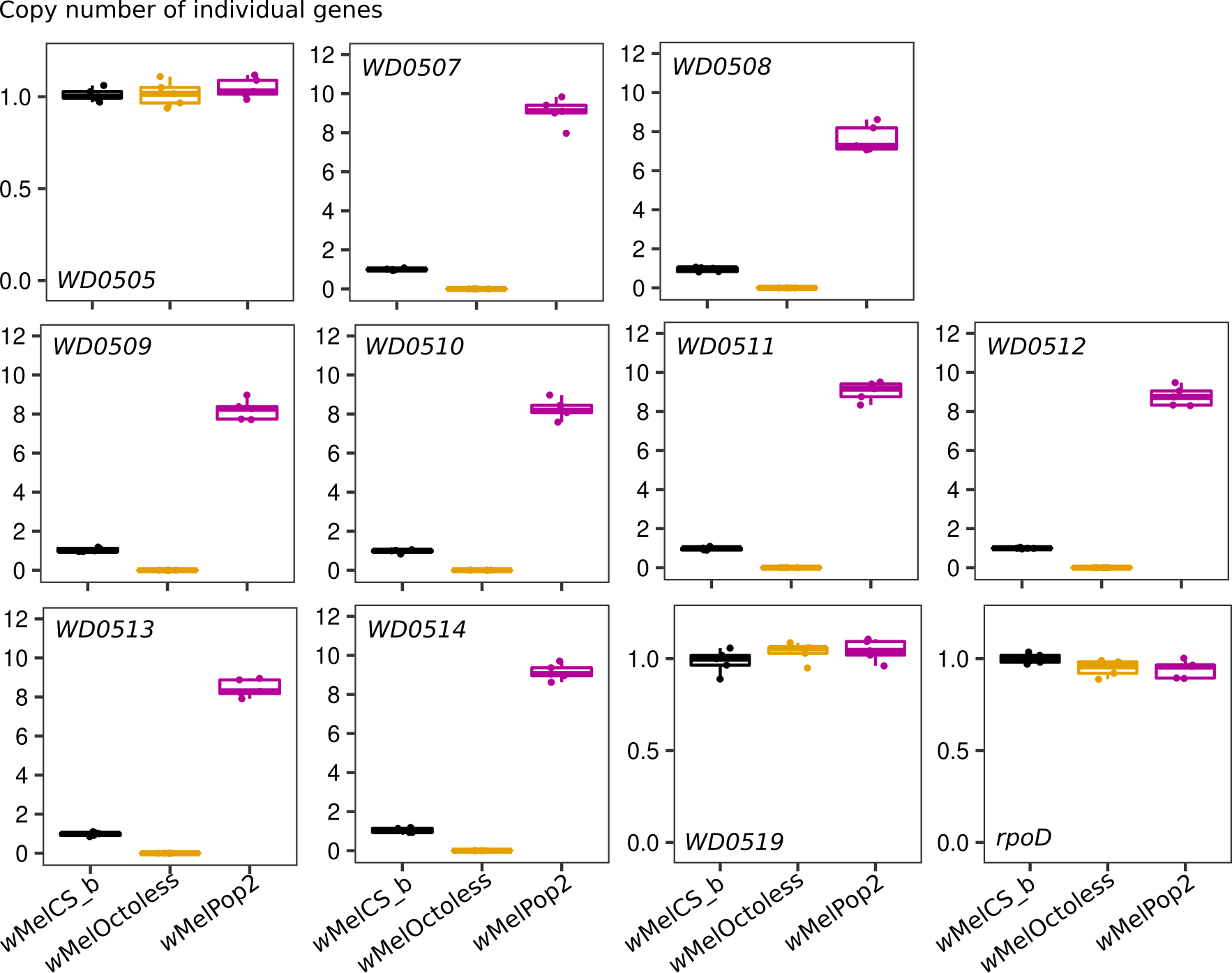

Supplement: S6 Fig — The amplification and deletion of individual Octomom genes (wMel loci WD0507–WD0514) was confirmed using qPCR in wMelPop2 and wMelOctoless, respectively. The copy number of three genes outside the Octomom region (wMel loci WD0505, WD0519, and rpoD) were also determined. Five females carrying wMelCS_b, wMelPop2, and wMelOctoless were used in the analysis. The copy number of wMelPop2 and wMelOctoless genes is relative to that of wMelCS_b. (TIFF) [file pgen.1009612.s006.tiff]

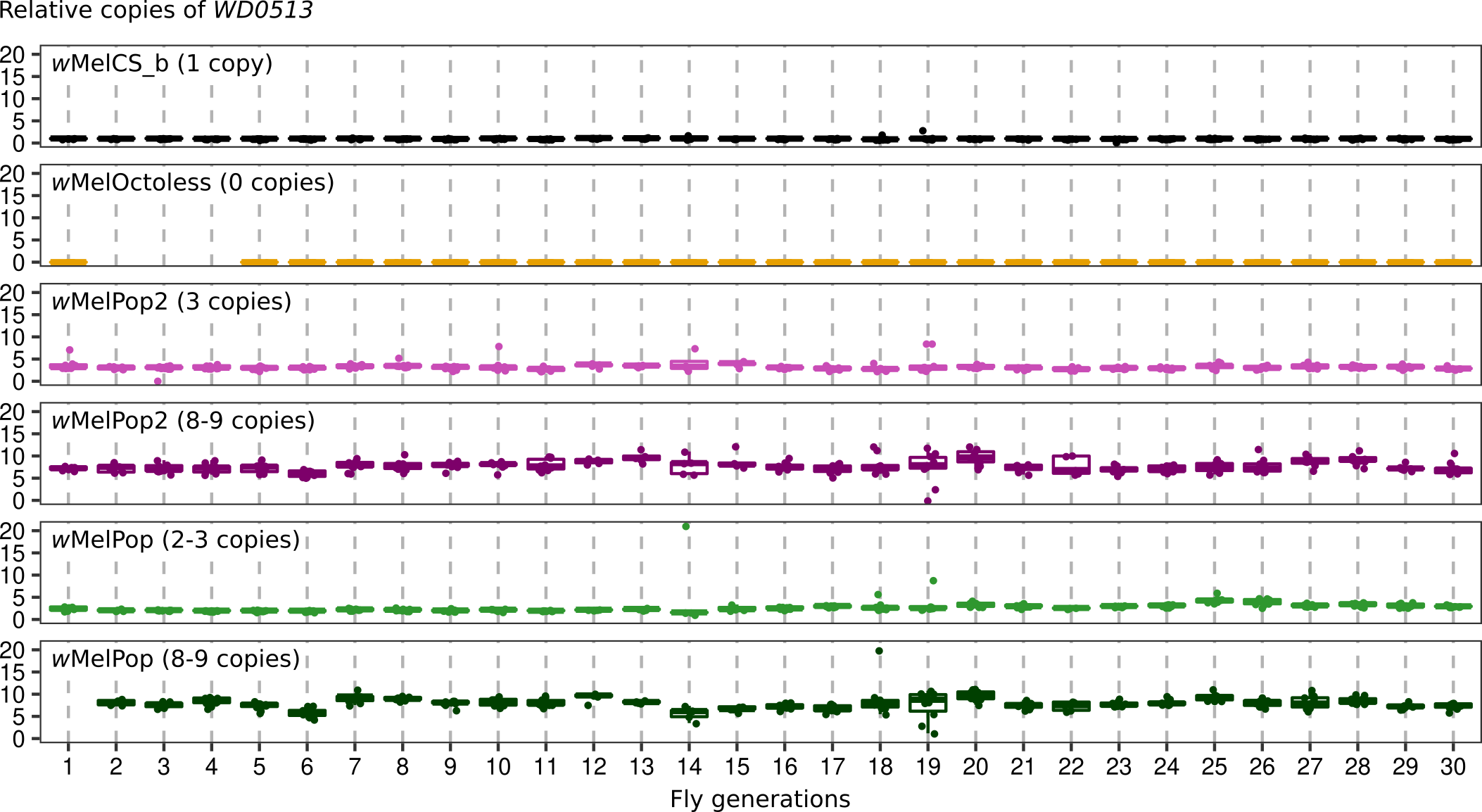

Supplement: S7 Fig — The relative copy number of genomic WD0513 in Wolbachia-carrying stocks throughout 30 fly generations. Each generation, 5–20 females were randomly collected for egg-laying for 3–4 days and used to determine the relative copy number of WD0513, as a proxy for the Octomom copy number. The progeny of a single female was used to set up the next generation. qPCR results were normalized to that of wMelCS_b, which has a single copy of Octomom per genome. (TIFF) [file pgen.1009612.s007.tiff]

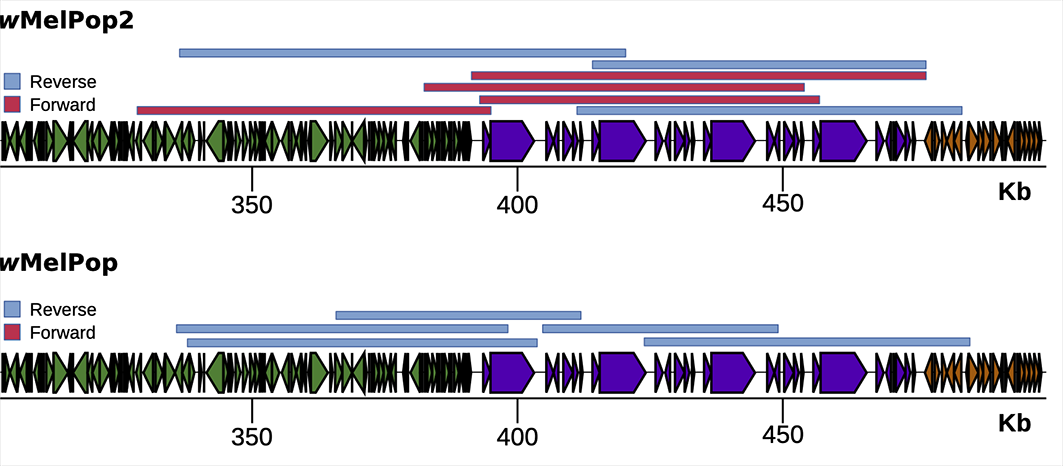

Supplement: S8 Fig — Oxford Nanopore MinION reads supporting the amplification of the Octomom region in tandem in wMelPop2 and wMelPop Wolbachia variants. We mapped wMelPop2 and wMelPop long reads (BioProject: PRJNA587443) to the the Octomom region in their genomes (Accessions CP046922.1 and CP046921.1, respectively) using minimap2 v2.17-r941 [48] and plotted the alignment summary (S7 Table) for illustrative purposes. (TIF) [file pgen.1009612.s008.tif]

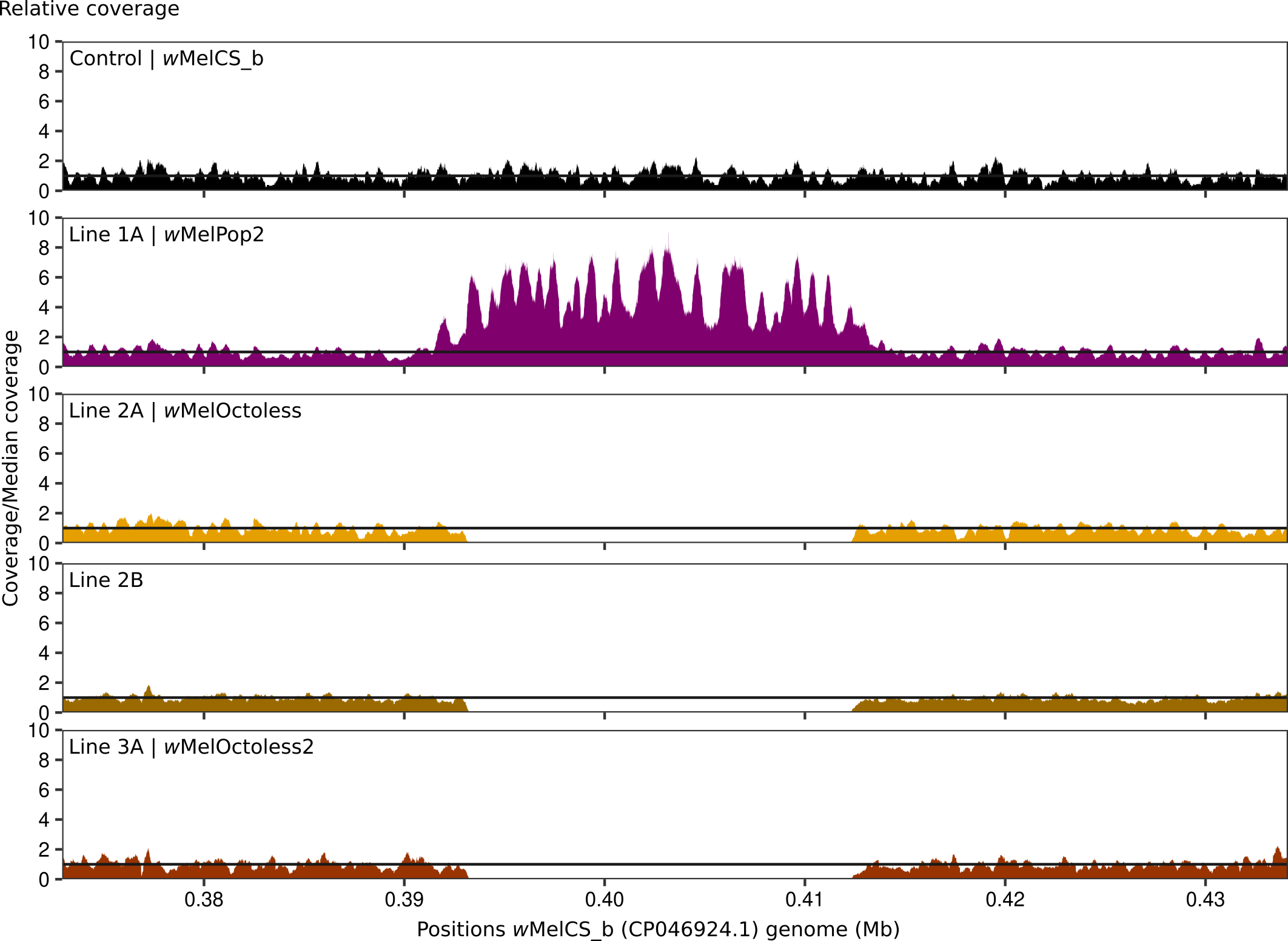

Supplement: S9 Fig — Relative coverage in the genomic region containing the Octomom region. As in Fig 2B, Illumina paired-end reads were mapped to wMelCS_b (GenBank: CP046924.1) genome, and the number of reads mapping to each position were normalized by dividing to the median coverage across the genome. Coverage information for wMelCS_b, wMelPop2 and wMelOctoless is also given in Fig 2B. We identified the deletion of Octomom as the cause of proliferation in lines 2B and line 3A (wMelOctoless2), as no other difference was found when compared to wMelCS_b. (TIFF) [file pgen.1009612.s009.tiff]

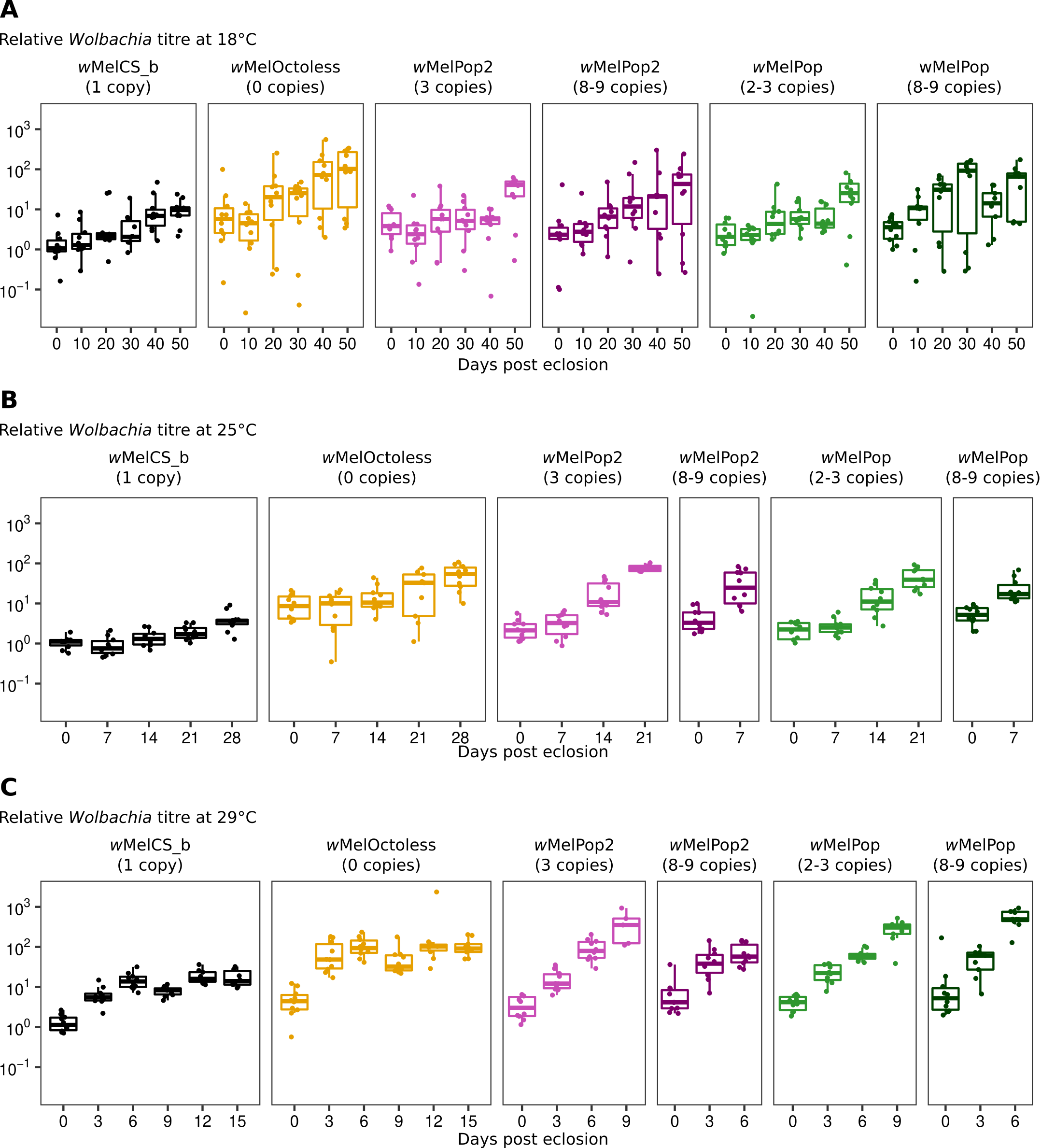

Supplement: S10 Fig — Time-course of relative Wolbachia titres in adults at 18°C (A), 25°C (B) and 29°C (C) with different Wolbachia variants. Replicate of experiment shown in Fig 3. Wolbachia titres were determined and analysed as described for Fig 3. (TIFF) [file pgen.1009612.s010.tiff]

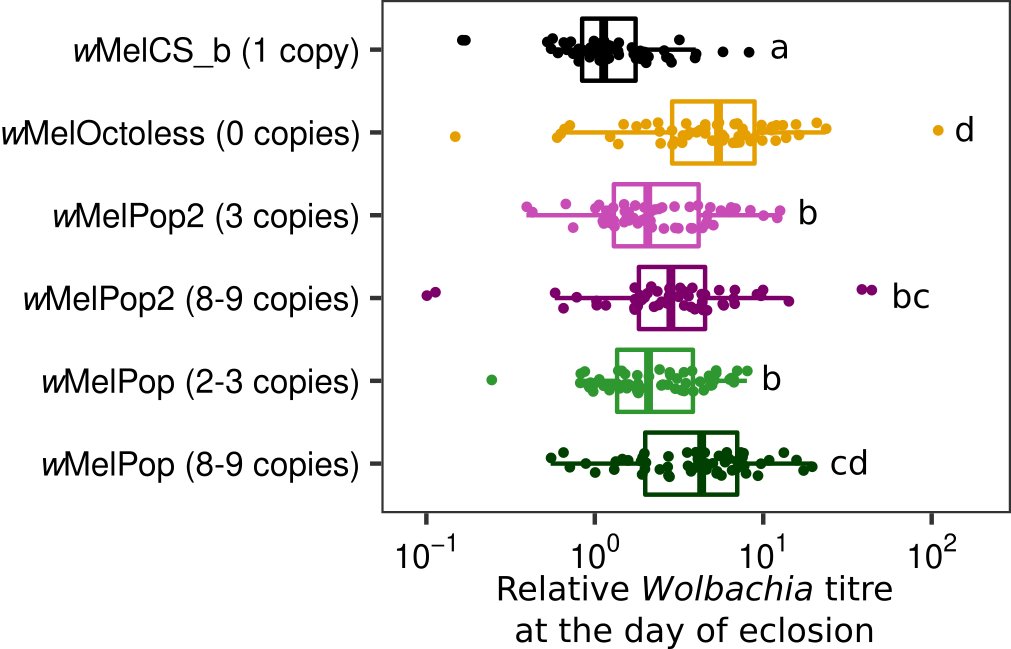

Supplement: S11 Fig — Relative Wolbachia titres on the day of adults eclosion. Males developed at 25°C were collected within 24 hours after eclosion for Wolbachia titre measurement using qPCR. Data used in this figure are also shown in Fig 3 and S10 Fig (time point 0). Letters represent significant groups after p-value correction. (TIFF) [file pgen.1009612.s011.tiff]

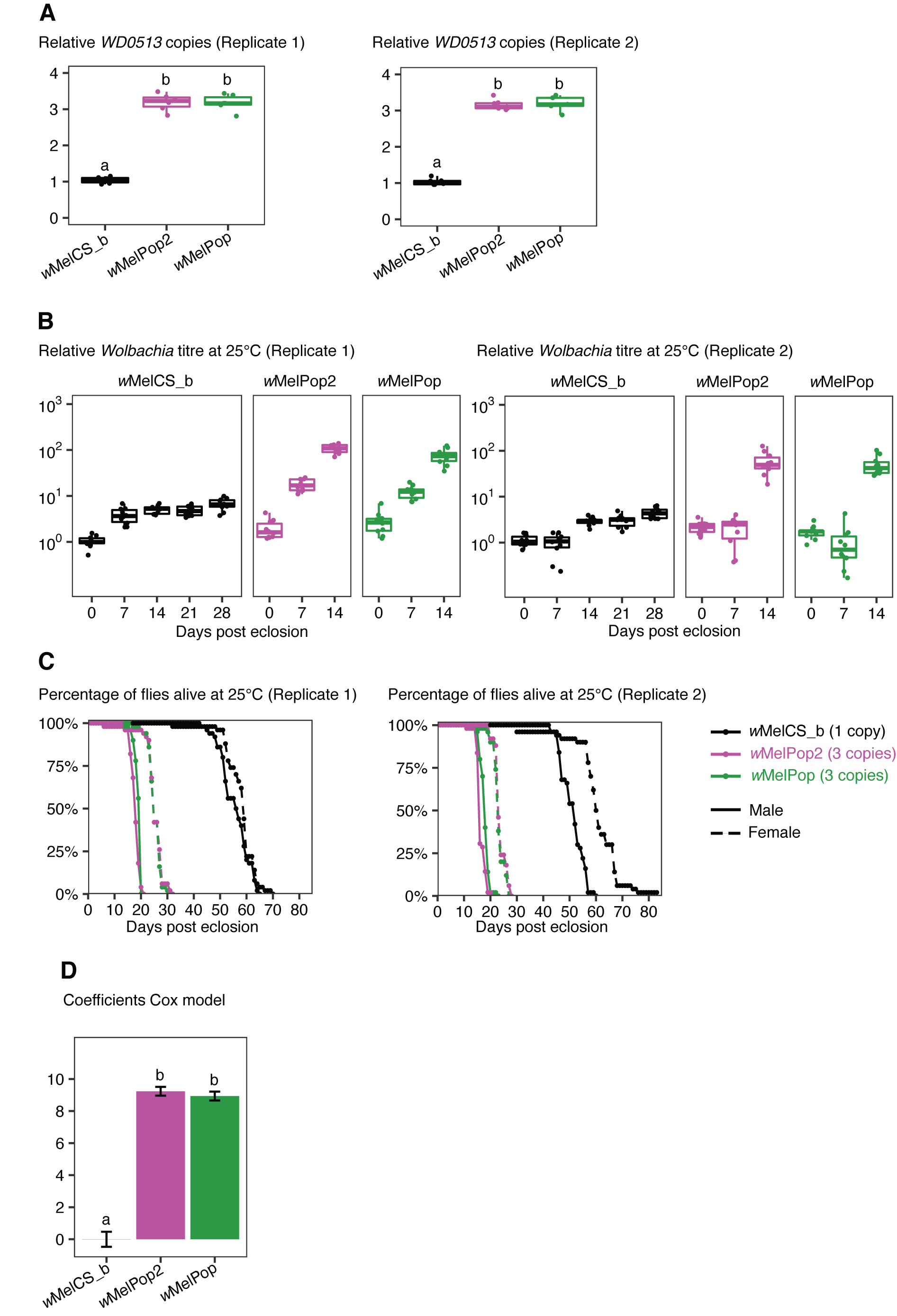

Supplement: S12 Fig — (A) WD0513 copy number of wMelPop2 and wMelPop in two experimental replicates. Using WD0513 as a proxy, the Octomom copy number of wMelPop2 and wMelPop was tightly controlled prior to phenotypic comparison. (B) Wolbachia relative titres at 25°C. The progeny of wMelPop2- and wMelPop-infected females carrying three copies of Octomom was used to set up the experiments. Males that developed at 25°C were collected upon eclosion, aged to specific time-points and used to determine Wolbachia titres using qPCR. Wolbachia titres were normalized to that of wMelCS_b-carrying flies collected on the day of eclosion. Proliferation rates of wMelPop2 and wMelPop were not different (p = 0.32). (C) Lifespan of males (solid lines) and females (dashed lines) flies at 25°C. Males were transferred to new vials every five days, while females every four days. (D) Coefficients of a Cox mixed model, representing the effect of wMelPop2 and wMelPop on the lifespan relative to wMelCS_b-carrying flies. wMelPop2 and wMelPop was equally pathogenic (p = 0.29). (TIF) [file pgen.1009612.s012.tif]

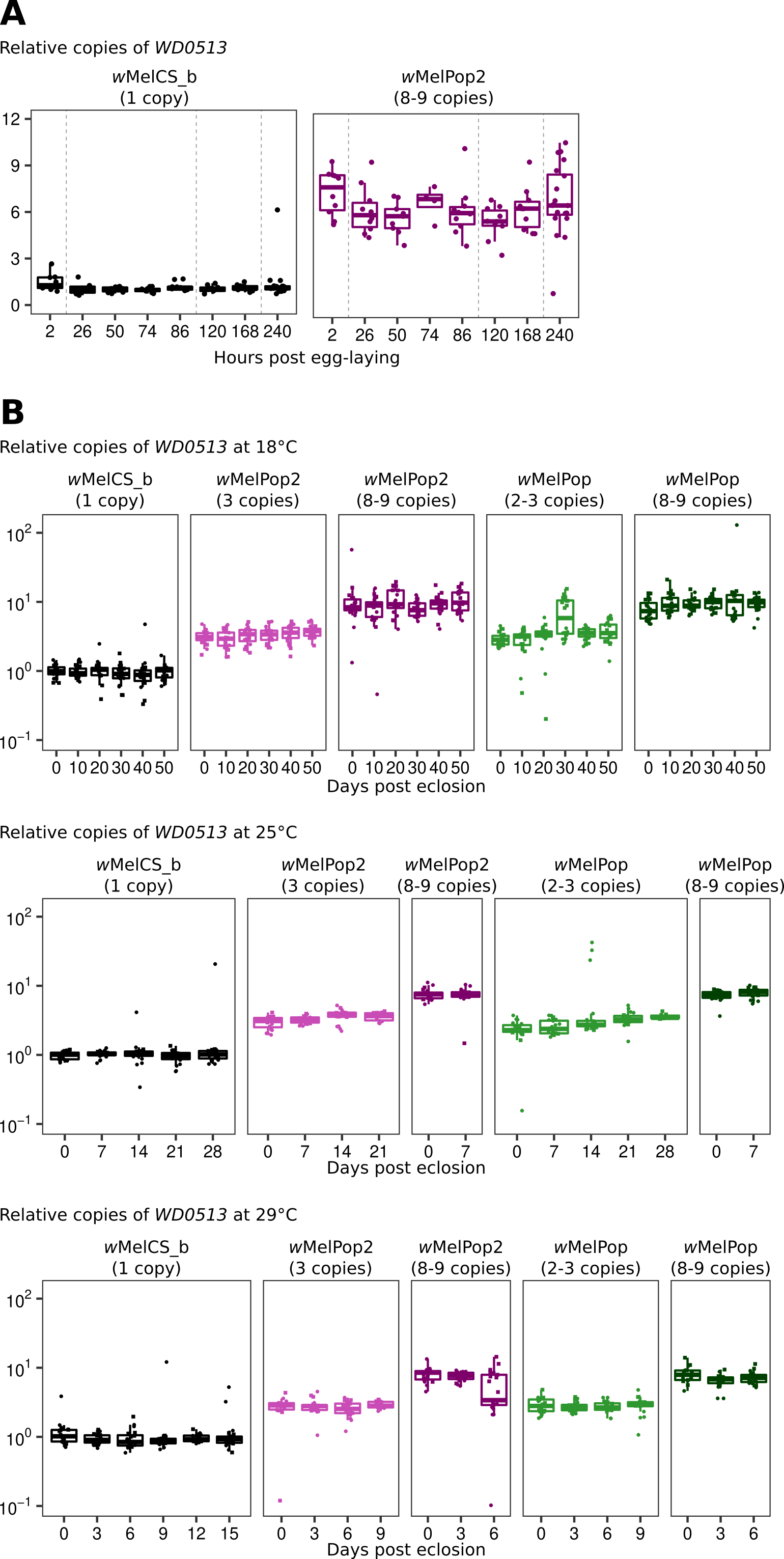

Supplement: S13 Fig — Relative copies of WD0513 throughout D. melanogaster development (A) and during adult life (B). WD0513 relative copy numbers were determined in samples shown in Fig 4 (for panel A) and Fig 3 and S10 Fig (for panel B). WD0513 copies were normalized to that of 0–1 old wMelCS_b-infected males. (A) Vertical dashed lines separate developmental stages (i.e. eggs, larvae, pupae, and adults). The x-axis is not in a continuous scale. (B) The two replicates are represented by different symbols. (TIFF) [file pgen.1009612.s013.tiff]

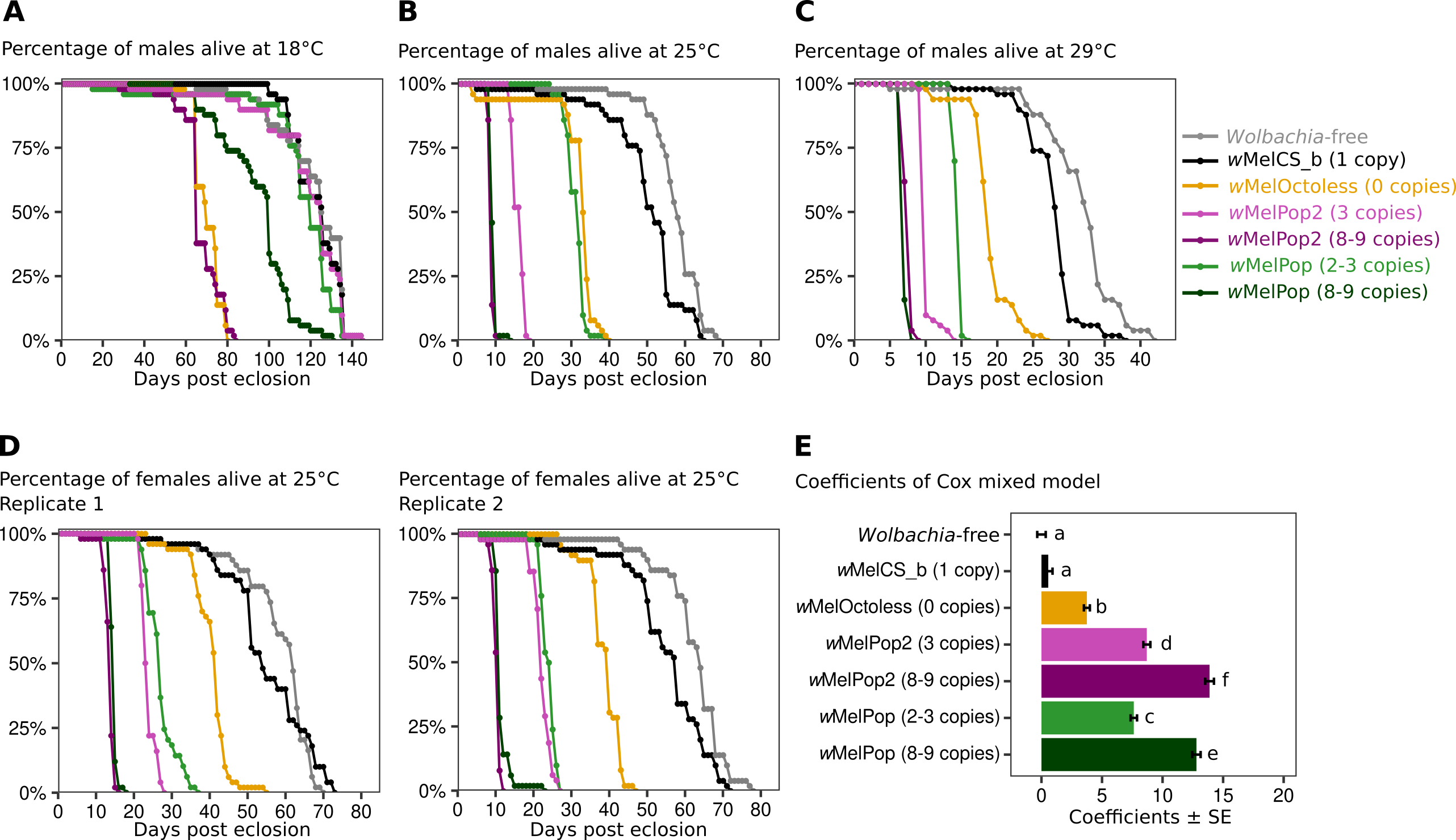

Supplement: S14 Fig — Lifespan of D. melanogaster males at 18°C (A), 25°C (B), and 29°C (C). Survivorship was determined as in Fig 5. This is a replicate of Fig 5. (D) Survival of D. melanogaster females at 25°C. Survival was determined as in Fig 5, except that females were transferred to new vials every four days. The experiment was performed twice. (E) Coefficients of a Cox mixed model of the lifespan of females relative to Wolbachia-free control. Both replicate experiments were pooled for statistical comparisons. Bars represent the standard error of the coefficient, and letters the statistically significant groups. (TIFF) [file pgen.1009612.s014.tiff]

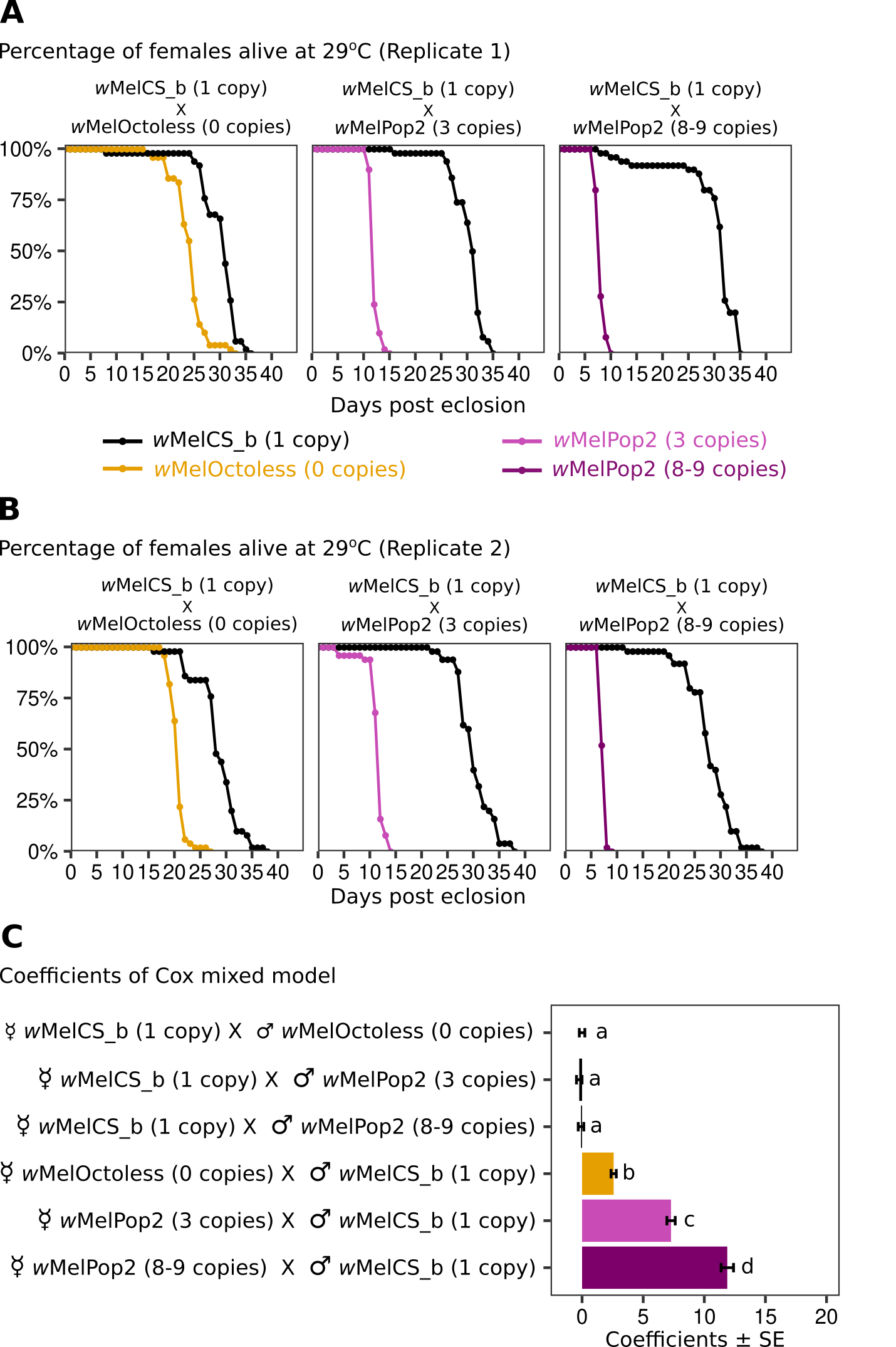

Supplement: S15 Fig — (A-B) Survival of D. melanogaster females at 29°C. Virgin wMelCS_b-carrying females were crossed with males carrying wMelOctoless or wMelPop2 (with 3 or 8–9 Octomom copies) and vice-versa. The resulting progeny developed at 25°C and was placed at 29°C after adult eclosion. The survival of 50 female progeny, which have the same genetic background but differ in Wolbachia infection, was determined per condition, per replicate. Females were maintained in groups of ten and transferred to new vial every four days. The experiment was performed twice. (C) Coefficients of a Cox mixed model representing the effect of the parental crosses on the survivorship of females. Significance was accessed after p-value correction for multiple comparisons, and significant groups are represented by letters. (TIFF) [file pgen.1009612.s015.tiff]

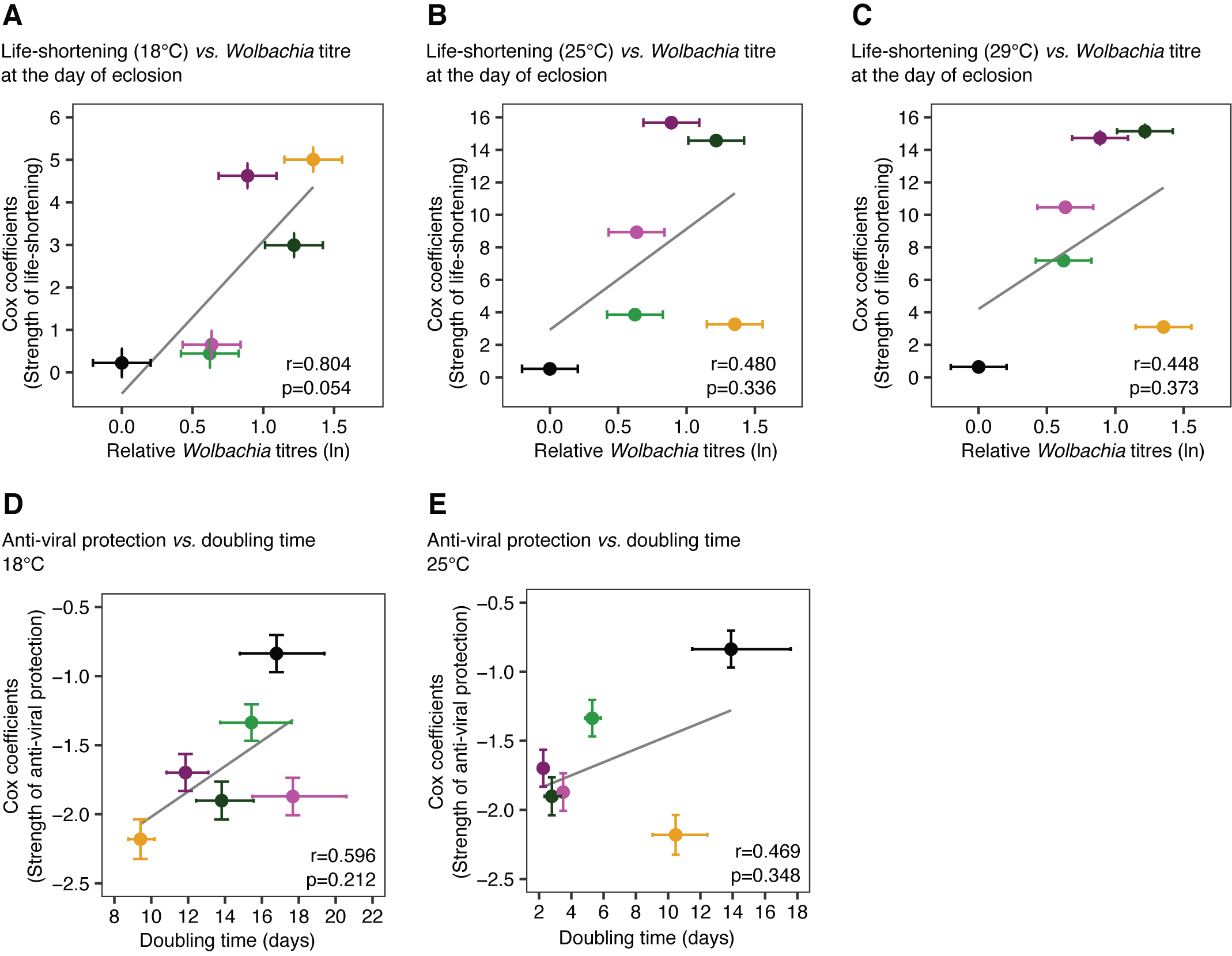

Supplement: S16 Fig — (A-C) Correlation between Wolbachia titre at the day of eclosion and the strength of life-shortening phenotype determined at 18°C (A), 25°C (B), and 29°C (C). The y-axis represents the strength of Wolbachia life-shortening phenotype (estimated using Cox mixed model shown in Fig 5). The x-axis represents the natural log of the relative Wolbachia titre estimated using a linear mixed model. Bacterial titres were normalized to that of wMelCS_b-infected flies (shown in S11 Fig). (D and E) The correlation between the strength of anti-viral protection and Wolbachia doubling time. The y-axis represents the strength of anti-viral protection (estimated using Cox mixed model shown in Fig 6). The x-axis represents Wolbachia doubling time in adults at 18°C (D), or 25°C (E) (shown in Table 1). The Pearson correlation coefficient (r) and its significance (p) are given in each panel. A grey line represents the trend (fit of linear regression). Error bars represent the standard errors of the estimates. None of these correlations were statistically significant and they complement correlations shown in Fig 5 and Fig 6. (TIF) [file pgen.1009612.s016.tif]

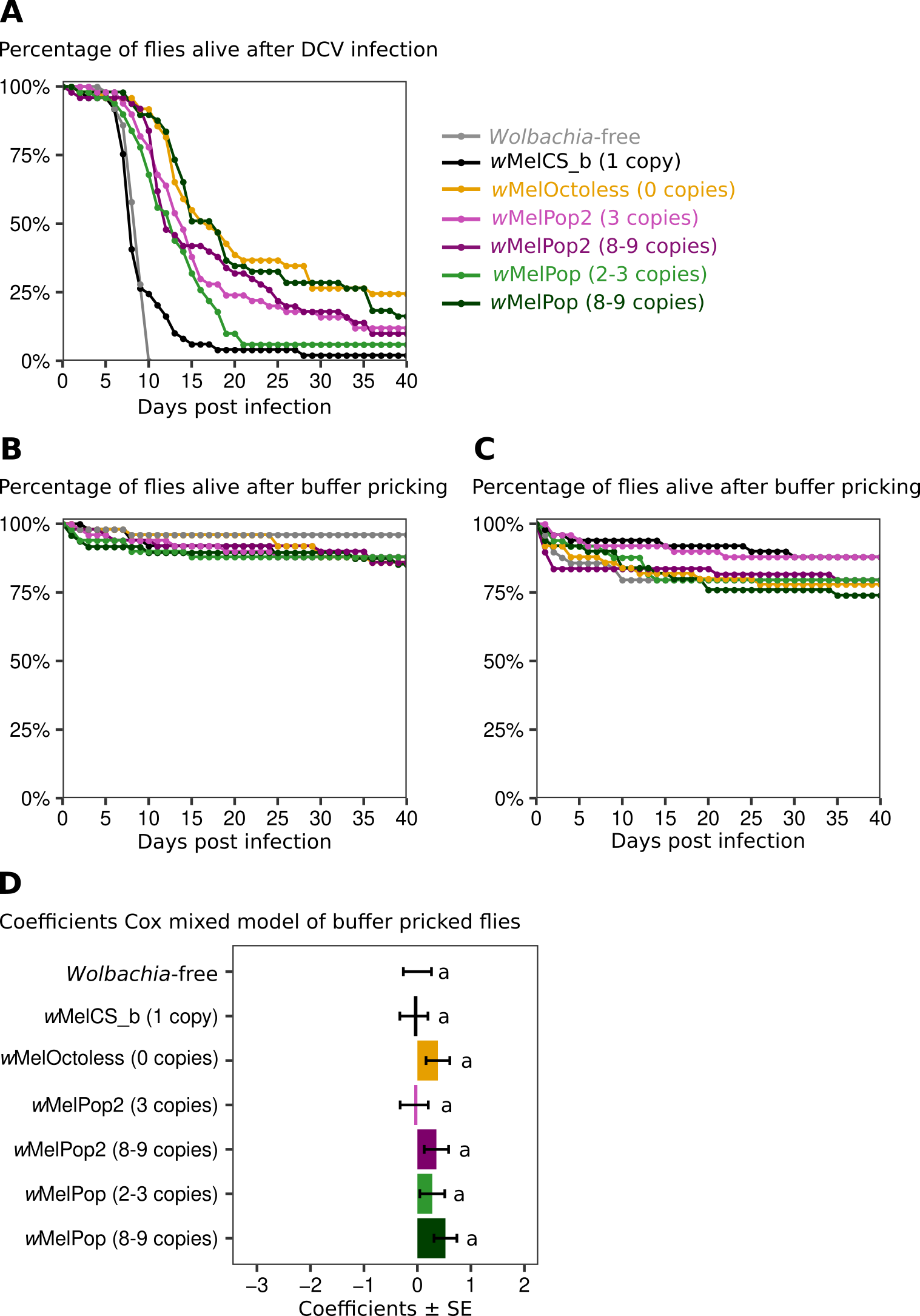

Supplement: S17 Fig — (A) Survival of males carrying different Wolbachia variants after a challenge with DCV (A) or a buffer solution (B and C). Fifty 3–5 days-old Drosophila males, per line, were pricked with DCV (109 TCID50/ml) or buffer and survival curves were determined at 18°C for 40 days. A is a replicate of Fig 6A, 6B and 6C are controls for these experiments. (D) Coefficients of Cox mixed models of buffer-pricked flies. Both replicates were pooled for statistical analysis. Bars represent the standard error of the estimate, and the letters the statistically significant groups after p-value correction. (TIFF) [file pgen.1009612.s017.tiff]
