# Supplementary material for: Forward genetics in Wolbachia: Regulation of Wolbachia proliferation by the amplification and deletion of an addictive genomic island
Source: PLoS Genet. 2021 Jun 18;17(6):e1009612. doi: 10.1371/journal.pgen.1009612 (PMC8244876; doi:10.1371/journal.pgen.1009612)
Supplement: Attachment — Submitted filename: Response to reviewers.docx [file pgen.1009612.s028.docx]

Forward genetics in *Wolbachia*: Regulation of *Wolbachia* proliferation by the amplification and deletion of an addictive genomic island

Duarte *et al.*

Response to reviewers (from PLOS Biology)

We are happy that both reviewers agree on the quality of the work and that the data supports the conclusions of the paper relative to the characterization of the new Wolbachia variants. The common comment from both reviewers is the fact that we do not have evidence that EMS mutagenesis worked by itself. As also noted by both reviewers we discuss this in the manuscript and we do agree that we not have evidence for this. Notwithstanding, the genetic screen as a strategy worked, we did isolate new overproliferative mutants, and the main interest of the paper is in their characterization.

**Reviewer #1: Duarte et al. describes a well conducted study on an experimental system to study the genetic basis underlying the replication of intracellular symbionts. In a Drosophila model, the authors explored the potential of a mutagenic agent, EMS, to induce mutations in the Wolbachia genome, and conducted a screen to identify three over-proliferating variants of the wMelCS_b Wolbachia strain. A series of experiments were conducted to phenotypically characterize two of these variants, and genome sequencing, used to characterize the genetic mutations underlying the phenotype. The results describe tandem amplification of an eight-gene region previously called "octomom" by the same group, and provide further data in support of the hypothesis that higher copy numbers are associated with higher proliferation, which in turns contributes to higher impacts on host fitness. The authors show that another mutant lacking the entire region also displays the over-proliferating**

**phenotype. The study also sheds further light on the complex links between symbiont proliferation, temperature and host development.**

**The paper will be of interest to the field of symbiosis as well as ongoing symbiont-based vector control applications. The conclusions are supported by the data; the authors conducted appropriate experiments to discount potential confounding effects such as mutations in the host or mitochondrial genomes. The novelty of the findings is unfortunately reduced by the previous papers on the same topic; originality primarily comes from the use of the mutagenesis agent EMS, but it is not actually demonstrated that EMS is the cause of the documented mutations (as acknowledged by the authors).**

**The eight-gene region is inherently unstable and mutants can arise spontaneously, at least in the lab where the fitness costs resulting from the symbiont over-proliferation may not be selected against - for instance, a previous study from the same authors has shown how easily one can select for higher or lower copy number of the region in the pathogenic wMelPop strain. There are also a few**

**previous examples of recent loss of the region in cell line models (fruit fly and mosquito). In light of this, it is not so surprising that the screening led to the discovery of 'octomom' variants rather than variant in genes located outside this region.**

We do not fully agree with the comments from the reviewer. We did show before that a previously identified overproliferative mutant had an amplification in the Octomom region and that the copy number of this amplification was unstable. However, it was unknown how easy would it be for de novo amplification from a one-copy “normal” Wolbachia. There was only one known case. Our work demonstrates that either by EMS or spontaneously this region amplifies relatively easily.

On the other hand, when we started this work there was one known case of loss of this region in cell culture, and a second case was published meanwhile. However, we were not screening for loss or gain of Octomom region. If that were the case it could be argued that it wouldn’t be a surprise to find it. We were indeed doing a forward genetic screen and, therefore, screening based on a phenotype, not on genotype. There was no expectation for loss of Octomom to be selected and therefore it was surprising.

This result was overall surprising to the Wolbachia community, as far as we can assess from the reactions and feedback after these data was presented. Please note that the group that first described Octomom loss in Wolbachia in Aedes cells and in Aedes mosquitoes did not associate this with overproliferation. On the contrary, this was used as evidence that the Octomom region was not involved in control of growth and that our group conclusions about Octomom amplification were wrong.

**The fact that many other genes likely contribute to Wolbachia proliferation, but none were identified here, would tend to suggest that EMS may not in fact have been the cause of the mutations described; a large screen of a thousand progeny such as this could have produced a similar outcome without the use of the mutagenic agent.**

As stated above we agree with this statement.

**The term "unbiased" to define this genetic screening is thus probably not appropriate, given the known instability of the region.**

Our screen was indeed unbiased since we were screening for phenotype not genotype. Although it can be argued that Octomom amplification was a possible hit (which happens in many genetic screens where some mutants are expected to be picked), the loss of Octomom mutant was not expected. Moreover, there could be other genetic elements unstable in the genome giving phenotypes too.

**The same rationale can be applied to the antibiotic treatment since we do not know if it substantially increased the probability for rare mutants to become fixed.**

We agree that we do not show the antibiotic treatment increased the probability of finding rare mutants. We do not make strong statements about this as conclusions, however, we had to described the protocol used and our rationale.

**Minor points**

**There is some repetition/redundancy throughout the text, e.g. lines 95: "mutant…variant", line 96: "causative…causing", line 111: "the variant…as starting variant").**

We corrected this and other redundancies in the text.

**197-198: Note that all of these SNPs/indels but one are also found in a recently sequenced wMel genome following transfer into Drosophila simulans (see table S2 in Costa Baião et al. 2021 ; https://www.biorxiv.org/content/10.1101/2021.01.29.428792v1.full). Therefore, they are most likely due to sequencing errors in the Wu et al. reference genome rather than true genetic differences.**

We make a reference to this in the discussion.

**474: specify fig 5E,F & G.**

We now specify the panels.

**584: change "ancestral" to "ancestor".**

We have corrected this.

**Reviewer #2: This is an interesting manuscript, and the experiments are thorough and of a high standard.  The standards of statistical analysis and the integration of experiments across temperatures is very good to see, given the known effects of temperature. The work is clearly a major undertaking, and the novel octomom deletion recovered is important and interesting - but my feeling was perhaps the approach less generally useful than the piece suggests.**

**Forward genetics for Wolbachia - clearly this is a holy grail of sorts, and the researchers do achieve apparent success in that de novo over proliferation mutants were recovered through the experiments.**

**However,**

**a)  the method is likely to only work well for this particular phenotype, due to the mixed population of Wolbachia within an individual - overproliferation mutants are like cancer cells in that they take over the population somatically. If we were interested in e.g. CI the approach would likely not work; what has been done is clever and innovative, but quite possibly particular to the study phenotype. The clever use of tetracycline might rescue the approach for other phenotypes, but this is not clear currently.**

As in the response to reviewer 1 we agree that the usefulness of the tetracycline is not clear from our data. But we do not claim that it is clear and merely explained our rationale.

The screen did work for overproliferation and part of our merit was to think of a phenotype and a screening strategy that would work in a forward genetic screen in Wolbachia. This could be applied to other symbionts.

The reviewer states that it may not work for other phenotypes, as CI. That may be true or not, it depends on someone to find a way to do it.

**b) do we know these mutations were the product of the EMS treatment? Of course, this does not matter in terms of genetic finding (a mutation is a mutation) - it matters only for the onward use of the EMS approach. It seems surprising that a mutation system that generally produces point mutations and microdeletions has produced wholescale copy number changes.  Of course, these things can occur sporadically with EMS. And of course, this is not the sort of experiment you do with non-EMS control - but I was left wondering if EMS did produce the mutations. Indeed, in Discussion the authors wonder the same thing.**

We answered to this comment above.

**The mutations found: The approach was successful in finding two overproliferation mutants, which is a strong success. However, they both pinpointed the existing region known to be associated with overproliferation, the octomom region. One of the two mutations is basically parallel to the previous - a copy number increase. The other is interesting - that deletion of the region wMelOctoless has a similar phenotype to increased copy number. In terms of phenotype-genotype link - this aspect is the novelty (that deletion as well as amplification produces the phenotype). It is also compatible with overproliferation being found in Aedes associated with deletion of this region- which is a very satisfying 'completion' to this story. Everything now ties together - no copy or too many copies produce this phenotype.**

As stated in the answers to reviewer 1 we agree that finding the Octoless mutant is the main novelty of this manuscript.

**I did wonder if there was any surprise that no point mutations were recovered for overproliferation using this approach - which is EMSs usual type of mutation.**

We discuss in the manuscript that point mutations were expected.

**Overall, therefore I was more interested and convinced by the mutations and phenotype than the method - which I am not clear is broad and not entirely convinced produced these mutations. I'd advise the authors to concentrate less on the method than the mutations - and not sell this as a novel general method for forward genetics - it could be, but I at least was not convinced that this was associated with EMS, nor that it would work outside of overproliferation.**

We agree that the new mutants and their characterization are the most important aspect of the manuscript. However, we did perform the first forward genetic screen in Wolbachia. Either EMS-induced or spontaneously generated, we isolated the first de novo mutants of Wolbachia with a phenotype. And this was useful to further understand genetic control of its growth. The method may not be ready to be applied to any phenotype of Wolbachia and we slightly changed the end of the abstract to reflect this. But we think the report of the strategy of the screen is interesting and important enough to inspire and be a starting point for other researchers.
